# Old-growth beech forests in Germany as cool islands in a warming landscape

**DOI:** 10.1038/s41598-024-81209-0

**Published:** 2024-12-05

**Authors:** Yojana Adhikari, Nadine Bachstein, Charlotte Gohr, Jeanette S. Blumröder, Caroline Meier, Pierre L. Ibisch

**Affiliations:** 1 Centre for Econics and Ecosystem Management, Eberswalde University for Sustainable Development, Alfred-Möller-Str. 1, 16225 Eberswalde, Germany; 2 Center of Methods, Faculty of Sustainability, Leuphana University, 21335 Lüneburg, Germany

**Keywords:** Climate crisis, Ecosystem functions, Land Surface Temperature, Microclimatic regulation, Normalized Difference Vegetation Index, Vitality, Forest ecology, Forestry, Climate-change impacts, Climate-change mitigation

## Abstract

The climate crisis seriously threatens Central European forests and their ecosystem functions. There are indications that old-growth forests are relatively resilient and efficient in micro-climatic regulation during extreme climatic conditions. This study evaluates five well-protected old beech forests in Germany, part of a UNESCO World Heritage Site. We examined temperature dynamics and vitality in core, buffer, and border zones during hot days from 2017 to 2023, using Landsat 8 and 9 imageries to assess Land Surface Temperature (LST) and Normalized Difference Vegetation Index (NDVI), alongside on-site Air Temperature (AT) measurements. Our findings reveal that all five forests were impacted by recent extreme heat events, with core zones remaining cooler and more vital, followed by buffer zones. Temperature-regulating patterns varied with landscape characteristics and the surrounding matrixes. We observed a site-dependent cooling effect of the forest interior that increased with higher LST. Our study highlights the value of old-growth forests and recommends increasing effective protection around mature forests, establishing corridors between isolated patches, and creating mosaics in managed landscapes that include unmanaged areas capable of developing into old-growth ecosystems.

**Supplementary Information:**

The online version contains supplementary material available at 10.1038/s41598-024-81209-0.

## Introduction

One of the most dangerous consequences of global warming which is associated particularly with increased frequency, intensity and duration of warmer summers and heat waves^1^. Heat poses threats to both ecosystem functioning^2^, and human health^3^. It affects human well-being also by harming agriculture^4^ as well as many other socio-economic activities^5^. It interacts with and intensifies other climate-related challenges, such as increased drought and water stress^6,7^. Central Europe has experienced several episodes of heat and drought extremes over the past two decades^8–11^. Since 2018, large parts of Central Europe not only suffered a record-breaking intense drought episode, but also witnessed simultaneous occurrences of climate extremes, encompassing warm and dry winters, significant decreases in precipitation, and heatwaves with record temperatures^12,13^. Germany was rated as the third most affected nation globally in the Global Climate Risk Index 2020, largely due to the impacts of heatwaves and droughts^14^. In fact, Germany is considered to be one of the climate hotspots in Central Europe^10^. Since 2003, Germany has experienced a significant increase in severe drought occurrences, notably during the summers of 2003, 2015, and 2018–2021. In Germany, past heat waves have led to a significant number of deaths, with current estimates perhaps still representing a gross under-estimate^15–17^. For health reasons alone, there is an urgent need to moderate extreme temperatures across Germany. For cities, the value of trees and parks as green infrastructure is increasingly understood and discussed^18^. At the landscape scale, forests represent an ecosystem-based solution to moderating extreme climatic and hydrological impacts, and should therefore be key elements in attempts to cope with increasing heat and drought in the coming decades^19^. However, the ecosystems that provide services related to cooling have themselves been affected by the heat waves and droughts of recent years^20^. In forests, there were serious growth reductions and a tree mortality increase across Central Europe^21–23^. During 2018 to 2020, approximately 7% of the forest area in Germany experienced a decline in forest vitality^24^. Similarly, between 2018 and 2021, over half a million hectares of German forests experienced tree cover loss^25^. The impacts of the weather extremes can be exacerbated by past and current forest management. Widespread conifer monocultures promoted bark beetle infestations, disease spread, and subsequent salvage logging impacted local climate and contributed to further fragmentation and warming of forest remnants in the cultural landscape^26^. Within a few years, Germany was plunged into a forest crisis that not only had an ecological dimensions, but also involved the traditional practices and reactions of the forestry industry^27–29^.

Many studies have indicated that the frequency and intensity of such extreme events in response to global warming are expected to become more common in the future^12,13^. Extreme weather events, such as the combined summer heat and drought in 2018, are likely to occur more often in the future and would cause forest cover decline. Therefore, it is ever more important to maintain or enhance the resilience and apply adequate forest management decisions that preserve meso- and microclimatic regulation capacities^30^. A crucial question is if non- or low- or high-intensity intervention forest management strategies are more appropriate to keep the ecosystems as healthy and functional as possible^28,31,32^. Therefore, assessments of ecosystem functioning in old-growth forests during climatically extreme periods are of high relevance to derive conclusions for adaptive forest and landscape management in the climate crisis. They can serve as reference and learning sites^32–34^.

Despite the evidence of a more effective microclimatic regulation in old-growth forests^33,35–37^, to date there are only limited studies in Central Europe that focus on microclimatic performance of these old forests at landscape level and during climatically extreme periods. One probable reason for this is that due to the long history of human land-use they have become very scarce; only 0.7% of European forests can be considered old-growth^38^.

**Fig. 1:**
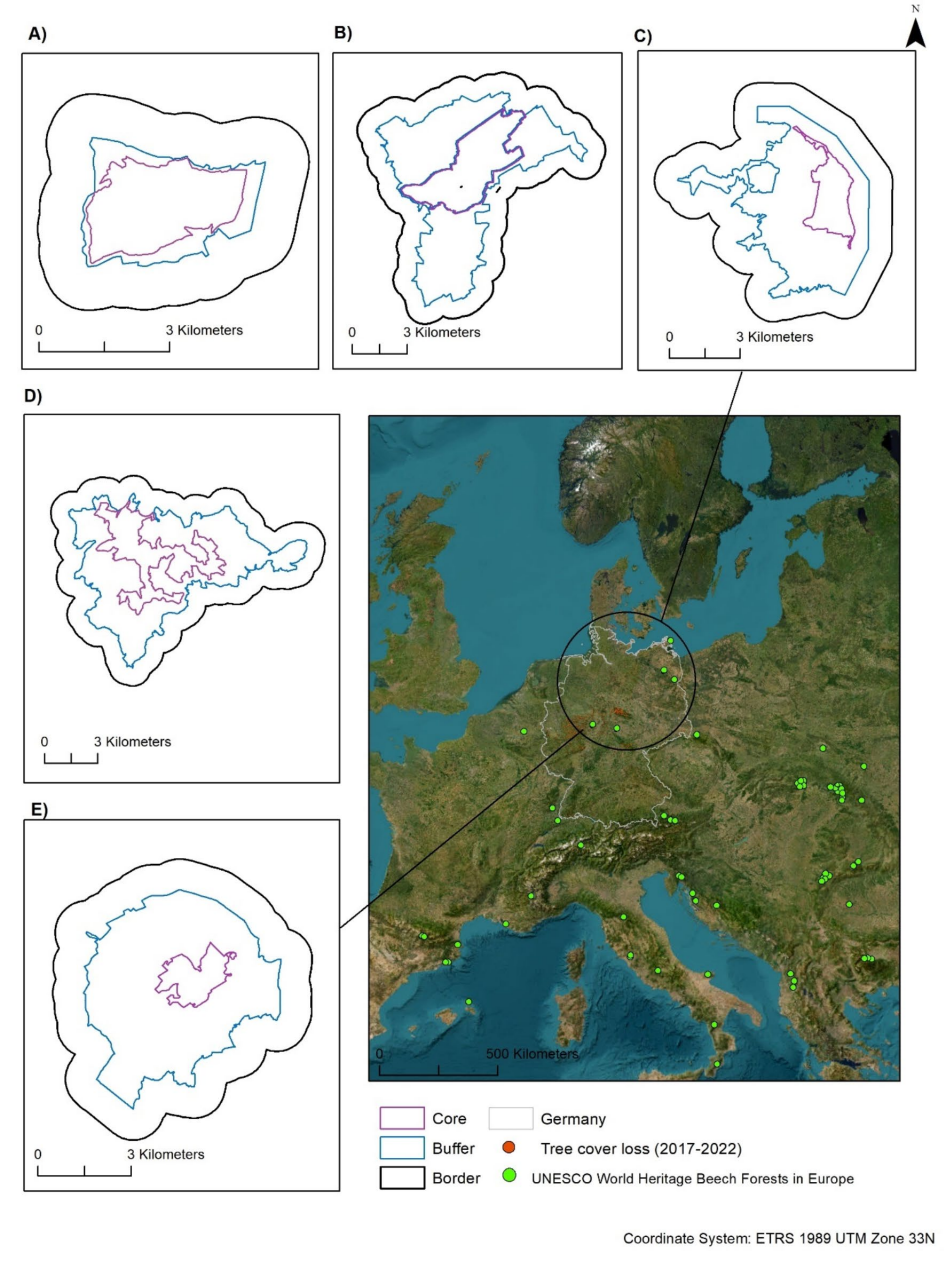
Location of the five investigated German component parts of the UNESCO World Heritage Site “Ancient and Primeval Beech Forests of the Carpathians and Other Regions of Europe”. The map in the right shows the location of the five component parts, and indicates tree cover loss in Germany between 2017 and 2022 in red. (source: Hansen et al.^49^), which was heavily influenced by heat and drought. On the left and above, shape and zoning of the five German component parts is illustrated with the core zone in pink, the buffer zone in blue and the border in black of **A**) Grumsin, **B**) Hainich, **C**) Jasmund, **D**) Kellerwald and **E**) Serrahn.

In this context, the UNESCO World Heritage Site devoted to the “Ancient and Primeval Beech Forests of the Carpathians and Other Regions of Europe” is a highly valuable resource. It has been initiated in 2007 in the Ukrainian and Slovak Carpathians, expanding to encompass German forest areas in 2011, and, from 2017 onwards, evolving into a pan-European project and the most complex serial World Heritage property in the world^39,40^. After a third extension in 2021, the site comprises 93 component parts across 18 countries^40^ (Fig. 1). The decision to concentrate on old-growth forests instead of general protected areas listed in World Database on Protected Areas (WDPA), is based on their unique ecological characteristics which enable a more comprehensive understanding of climate impacts and potential mitigation strategies. These forests unfold the story of the post-glacial spread of European beech (*Fagus sylvatica*), an ongoing ecological phenomenon now heavily influenced by contemporary climatic changes^38,41^, while general protected areas contain not only old, but also heavily managed and changed forests. This unique setting of well-protected, relatively old beech forests offers special conditions for studying their importance, not only as anchors of old-growth biodiversity, but also in terms of their landscape ecological functions. Furthermore, the rarity of these ecosystems also emphasizes the need for dedicated research focused on these types of forests. In Germany, there are five component parts covering different beech forest types from the Baltic Sea to mid-elevation mountains: Grumsin, Hainich, Jasmund, Kellerwald, and Serrahn. These five old-growth forests, while showing traces of different human use in the past centuries, showcase mature beech forests with distinct historical utilization and varying landscape contexts (Fig. 1; Table 1).

**Table 1:** Characteristics of the five component parts of the study area. [Grumsin^39,40,42^; Hainich^39,41,42^; Jasmund^39,41,42^; Kellerwald^39,40,43,44^; Serrahn^39,40,42^; compare fig. S4]

Component part	Size [ha], and Eleva- tion [m a.s.l.]	Landscape character- istics (see also Figure S4)
Grumsin (site A)	core:590, buffer:274; 76 to 139	Combination of water bodies and forest; surrounded by agricultural landscape matrix
Hainich (site B)	core:1573, buffer:4085; 290 to 490	Largest unmanaged deciduous forest area in Germany, forested mountain range surrounded by largely open matrix landscape; pastures and agricultural areas
Jasmund (site C)	core:492.5, buffer:2510.5; 0–131	The most extensive continuous beech forest along the Baltic coast, but relatively isolated; Baltic Sea as special and dominating matrix feature, agricultural areas to the west
Kellerwald (site D)	core:1467, buffer: 4271; 245–626	Beech forests with relatively undisturbed ecological and biological processes under oligotrophic and mesotrophic conditions; characterized by relatively fragmented forest landscape
Serrahn (Site E)	core: 268, buffer: 2568; 67–124	The structurally richest lowland beech forests in Europe; embedded in a matrix dominated by pine-stands

In this paper, we investigate the role of these German component parts of world heritage beech forests in micro and meso climate regulation, focusing on temperature cooling, especially during hot days of the recent extreme years. The cooling effect refers to the ability of forests to lower ambient temperatures, especially during hot days, through shading, evapotranspiration, and reduced solar radiation^45^. Within dense forest canopies, this is measured as the difference between land surface temperature (LST) and ambient air temperature (AT), reflecting the direct cooling impact of the canopy^46^ or as the difference between macroclimate (regional weather data) and local microclimate (within the forest)^47^. Additionally, we also compared hot day LST between different zones—core, buffer, and border zones, and component parts—highlighting the gradient in temperature regulation.

The aim of the study is to: (i) map temperatures in core and buffer zones, with more or less open stands and non-forest areas; (ii) investigate the forests’ performances in coping with the elevated temperatures in recent years; forest performance in this context relates to how well these forests cope by regulating itself during the crisis also includes cooling capacity and greenness overtime and (iii) evaluate whether the temperature regulation patterns of the five component parts exhibit any similarities and allow for general conclusions for management. Although this study focuses on five specific forests in Germany, the lessons learned here regarding the relationship between forest conservation and ecosystem health could apply to other temperate forest regions with similar climatic and ecological characteristics. However, it is important to be cautious when generalizing these results to all forest types, particularly those in different climatic conditions or under varying management regimes.

## Materials and methods

### Study area

Our research encompasses the five German components of beech forests recognized by UNESCO in 2011 as “Ancient and Primeval Beech Forests of the Carpathians and Other Regions of Europe”^39,48^ which are all embedded within larger designated protected areas (Fig. 1) : Hainich National Park (Thuringia), Kellerwald-Edersee National Park (Hesse), Jasmund National Park (Mecklenburg-Western Pomerania), Serrahn in the Müritz National Park (Mecklenburg- Western Pomerania), and Grumsin in the Schorfheide-Chorin Biosphere Reserve (Brandenburg).

Each of the five component part has a distinctive land-use history and local characteristics making it unique and irreplaceable; Grumsin has been a hunting ground of nobles and politicians for centuries, and now is part of the core zone of a UNESCO Biosphere Reserve; Hainich, once a military training ground of the German Democratic Republic, became a National Park in 1997; Jasmund on the island of Rugania has been protected since 1929 and became a National Park in 1990; Kellerwald was a royal hunting ground and became first a nature reserve (1935) and then a National Park (2000); Serrahn also has a history as a hunting ground for the regional nobility and became part of a National Park in 1990^40^. In these protected, (more or less) old-growth forests, only small areas, if any, have escaped more or less severe use and degradation in the past; they cannot be considered ‘primeval’. In Jasmund, for example, only the forest on the steep chalk cliffs was never managed, but large parts of today’s national park were heavily modified until the first half of the 20th century, including clear-cutting and conifer plantations^41^.

At these World Heritage component parts, forestry interventions have ceased in core and buffer zones for at least 20–30 years, though core zones have had legal protection for longer. The size of the core and buffer zones were already established prior to this study and were not defined by our research^40^. To further investigate any potential microclimatic changes outside the component parts, we created an additional area extending 1 km beyond the outline of the buffer zone, referred to as the *border zone.* It is essential to recognize that different threshold values could influence our results. Although a larger border zone might capture additional microclimatic variations, it could also introduce confounding factors from adjacent land uses or environmental conditions that do not directly relate to the component parts. All the beech forests studied are located at relatively low altitudes, mostly well below 500 m, and do not have any particularly pronounced topographical extremes that could strongly influence the surface temperature (Fig. S5). The different location (component parts) are accounted for as random effects in the linear mixed effect models. Therefore, the small differences in elevation are accounted for in the analyses.

### Satellite imagery

Our analyses were based on three pre-processed datasets to explore Land Surface Temperature (LST), the Normalized Difference Vegetation Index (NDVI) and land cover (Table 2). LST and NDVI were processed from the Landsat 8 and 9 mission, a series of satellites that was launched in 2013 and 2021 by the United States Geological Survey and the National Aeronautics and Space Administration (NASA) and provides high quality imagery of earth’s surface at a 30 m resolution^50^. Land cover data was obtained from the CORINE (coordination of information on the environment) dataset within the Copernicus Land Monitoring Service^51^.

**Table 2:** Properties of remotely sensed data sets used.

Product	Sensors	Spatial resolution	Temporal coverage	Number of images	Sequence and Time of recording
LST	Landsat 8 and 9	30 m	01.01.2017 to 30.08.2023	237	Time series, 16 days interval, ~ 11:15 am
NDVI	Landsat 8 and 9	30 m	2017 to 2023 (May, June, July, August, September)	145	Time series, 16 days interval, ~ 11:15 am
Land cover	Copernicus	100 m	2018	1	~ 11:15 am

### In-situ measurement of air temperature

A general cooling pattern of the forest interior was observed while comparing the satellite derived LST and ground measured AT^46,52^. In order to quantify the eventual difference between LST and AT, we carried out in-situ measurements of AT using altogether 69 data loggers (Onset HOBO-Pendant) distributed over the core zones of four component parts (Grumsin, Hainich, Kellerwald and Serrahn) (Fig.S3). We could not include Jasmund due to technical reasons. The selection of the location of the data loggers based on location of the dense old-growth forest. We compared LST and corresponding AT using data logger, in the core zones of each of the four component parts (Table 3). The loggers recorded temperatures every ten minutes at 1.3 m above the surface. As the LST at the were recorded continuously at ~ 11:15 am, we used the mean AT of values measured at 11:10 and 11:20 am for comparing both. Some loggers were excluded completely due to unavailability of corresponding LST datasets, damage, theft or placement problems and we also excluded logger readout days resulting in readings from 69 data loggers.

**Table 3:** Observation period used for comparing landsat LST and AT from data loggers.

Component parts	Observation period of AT and LST	Number of data loggers used for AT	Number of observation days
Grumsin (site A)	2022-05-11 to 2023-04-14	17	72
Hainich (site B)	2022-06-02 to 2023-06-12	11	40
Kellerwald (site D)	2022-05-31 to 2023-05-22	18	54
Serrahn (site E)	2022-05-10 to 2023-06-27	23	69

### Data analysis

Our LST analysis of extreme weather conditions was focused towards recognizing ‘hot days’ between 2017 and 2023 using Google Earth Engine^53^ platform. According to the German Weather Service (DWD), a ‘hot day’ is defined as a day when the air temperature exceeds 30°C^54^. In our study we defined hot day as one where the maximum LST reached or exceeded 30°C in any forest pixel in Germany. We acknowledge that, in forest ecosystems, air temperatures are generally slightly lower than LST. Therefore, while it is likely that there will be more days with LST surpassing 30°C than days where air temperatures do so, this threshold effectively captures the essence of extreme temperature days. Hence, it is suitable for our analytical approach. We compared the results with LST below 30°C to account for uncertainties in the results. To enhance result accuracy, areas with cloud cover below 11% were excluded from our consideration. The resulting LST time series was reduced to a single image with the per-pixel mean of all identified hot days. Our LST datasets are a suitable indicator of temperature in the forest due to the close resemblance with air temperature when investigating the impact of forest cover on local temperature even at different zones; tropical, temperate and boreal^55^.

This study specifically examines forest ecosystem functions to assess forest health. Our selection of NDVI and LST as indicators is based on Pettorelli et al.^56^. Many studies shows NDVI as a reliable proxy for assessing vegetation greenness or vitality and also a key predictor variable for understanding the effect of temperature on forest coverage^57–60^. Furthermore, recent studies conducted in Germany, including those by Mann et al.^26^ and Gohr et al.^19^ have successfully used remotely sensed LST and NDVI as indicators for evaluating forest health. For NDVI, we averaged the “greenest” pixels i.e., highest NDVI value during the summer months (May-September) between 2017 and 2023, since heat stress might result in distorted pictures of vegetation^19^. We used the near-infrared (NIR) band of Landsat series with a temporal resolution of 16 days and spatial resolution of 30 m. In Landsat 8 and Landsat 9, band 5 corresponds to the NIR band, and band 4 corresponds to the R band for assessing the NDVI (Table 2). The value of the NDVI ranges from − 1 (non-vegetated areas) to 1 (densely vegetated areas).

Furthermore, to calculate NDVI change we subtracted the computed NDVI of 2017 from that of 2023. We used an open-source code by Ermida et al.^61^ to calculate LST and NDVI. We used global forest change data from Hansen et al.^49^ to determine tree cover in 2017 and tree cover loss (2017–2022). We defined forested areas as “*tree cover ≥ 30%*”. We classified Forest cover as “*broad-leaved*” and “*coniferous*” using the Copernicus CORINE Land Cover dataset. For data acquisition, processing, and statistical analysis, we utilized the following software and platforms: Google Earth Engine^53^ for acquisition and processing of LST, NDVI and land cover data, R version 4.2.0 for statistical analysis^62^, and ArcMap version 10.8.2 for geospatial visualization and mapping^63^.

We employed three linear mixed-effect models (Table 4) using lme4 package in R to explore (i) if locations and forest cover types and NDVI affect overall LST for both hot (LST > 30 °C) and cooler (LST < 30 °C) days, (ii) if the influence of NDVI on LST changes is related to specific zonation, and (iii) if LST responded differently over the years in different zones. To determine if there were significant differences in NDVI between the zones, we performed a Kruskal-Wallis test followed by post-hoc pairwise comparison between different zones.

**Table 4:** List of linear mixed-effect models employed. *Zone* represents core, buffer and border area of each forest, *location* represents five component parts, *Forest cover* indicates the type of forest vegetation present, classified as broad-leaved or coniferous, *Year* spans from 2017 to 2023.

Model	Equation
i	LST ~ Forest Cover * Location + NDVI + (1|Location/Zone)
ii	LST ~ NDVI * Zone + Year + (1|Location/Year)
iii	LST ~ Year * Zone + NDVI + (1|Location/Year)

We used Spearman’s correlation test to assess the relation between LST and AT. The time series analyses for LST and NDVI from 2017 to 2023 were executed for the forest areas within core, buffer, and border zones categorized based on forest types—coniferous, broad-leaved.

## Results

### Temperatures and temperature changes in and around protected old-growth forests

Our results show in total there were 237 cloud free hot days between 2017 and 2023. The average per-pixel mean for these 237 days shows that LST exceeded 36 °C in all five component parts (Grumsin: 43.2 °C, Hainich: 40.0 °C, Jasmund: 36.1 °C, Kellerwald: 40.3 °C, and Serrahn: 39.2 °C) and all of them were measured in the border zones. Based on the linear-mixed effect model i, Jasmund remained coolest (β = −4.38, *p* < 0.001) and Grumsin was warmest (intercept, *p* < 0.001) during hot days. The explained variance for this model i was for fixed effects (forest cover, location, NDVI) R^2^ = 0.481, and for random effects (location and zonation) R^2^ = 0.749. The coniferous forests were slightly warmer (β = 0.55, *p* < 0.001) than the broad-leaved ones (Table S1).

In a second model ii, we found that forests in the core zone were cooler than the buffer zone (β = 4.16, *p* < 0.001), which in turn was cooler than the border zone (β = 5.59, *p* < 0.001) for all years between 2017 and 2023 (Table S2). The explained variance for this model ii was for fixed effects (NDVI, zonation, year) R^2^ = 0.415, and for random effects (location and year) R^2^ = 0.891.

**Fig. 2:**
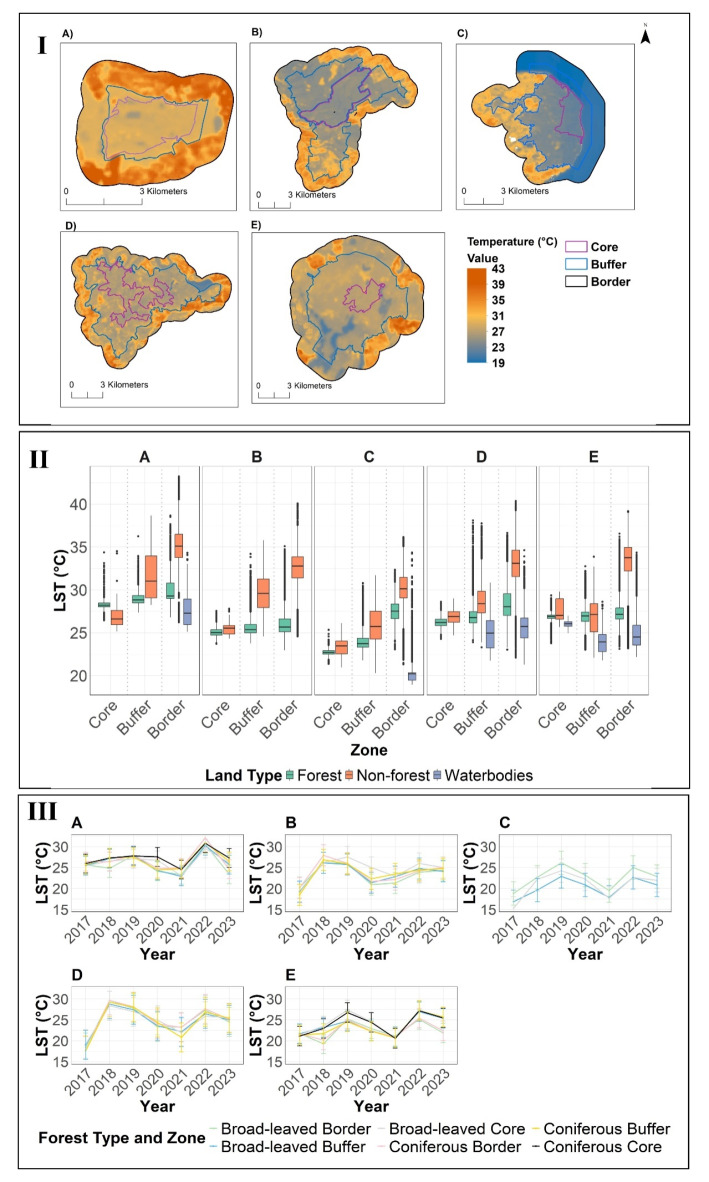
Hot-day Land Surface Temperature in German World Heritage Beech Forests between 2017 and 2023; **A**) Grumsin, **B**) Hainich, **C**) Jasmund, **D**) Kellerwald and **E**) Serrahn using Landsat 8 and 9 as the per pixel mean of hot days (**>** 30°C). (**I**) Maps of recent LST (average of hot days between 2017–2023) for the core, buffer and border zones, (**II**) Recent LST (average of hot days between 2017–2023) for the core, buffer and border zones in forests, non-forests and water-bodies, and (**III**) LST time series as a mean of hot days (**>** 25°C) per year for the core, buffer and border zones and distinguished by forest types. Forests defined as tree cover greater than 30%. A ‘hot day’ in this study was defined as one where the maximum LST reached or exceeded 30 °C in any forest pixel in Germany.

In the forested area of Grumsin and Hainich, the average LST on hot days of overall years gradually increased from the core to the buffer zone, with a more pronounced rise observed in the border zone (Fig. 2II). Non-forest areas within Grumsin’s core were 1.5 °C cooler than the forests (Fig. 2II). Among all components, Grumsin forests recorded the highest average LST on hot days across all zones (core: 28 °C, buffer: 29 °C and border: 29 °C) (see Fig. 2II). In contrast, the coastal Jasmund forest exhibited cooler temperatures across all zones (core: 22.5 °C, buffer: 24 °C, and border: 27 °C) and the components parts. However, the mean hottest day LST differences between the border forests and both the core and buffer forests were more pronounced in Jasmund in comparison to the other component parts (see Fig. 2II).

Kellerwald forests displayed an average hot-day LST of 26 °C in the core, going up to 27 °C in the buffer zone, and peaking at 30 °C in the border zone (Fig. 2II). Patches of warm temperatures were noticeable in the south-western region of the buffer zone (Fig. 2I).

In the forested area of Serrahn, the average hot-day temperatures in the core and buffer zones were almost the same, and some parts of the buffer zone were even cooler than the core (Fig. 2II). Also, the mean hot-day LST differences between the core, buffer, and border forests were at the same level (27 °C) (Fig. 2II).

The time series data displayed above indicates the average yearly LST for days exceeding 25 °C. This temperature threshold of 25 °C was selected because not all forests experienced days with temperatures reaching 30 °C at the beginning of the study period (Fig. 2III).

Overall, we observed an increasing pattern of mean hot day LST after 2017 in all component parts. Our results indicate that the years 2018 (β = 4.61, *p* < 0.001), 2019 (β = 5.97, *p* < 0.001), 2022 (β = 6.57, *p* < 0.001) and 2023 (β = 4.64, *p* < 0.001) were significantly warmer years, while 2020 and 2021 did not show statistically significant results (Table S2). Based on a third model iii, the significant interactions between year and zone type indicate that the effect of zoning on LST changes over time indicating the temporal factors in moderating local temperatures (Table S3). The explained variance for this model iii was for fixed effects (year, zonation, NDVI) R^2^ = 0.427, and for random effects (location and year) R^2^ = 0.898.

In Grumsin, from 2017 to 2020, the core and buffer zones had fewer temperature fluctuations, while border zones experienced steep rises and falls (Fig. 2III). For instance, broad-leaved border forests displayed average hot-day LST were 25.5 °C, 25 °C, 27.5 °C and 24 °C in 2017, 2018, 2019 and 2020 respectively. The year 2021 was cooler than 2017 (below 25 °C), the 2021 average hot-day LST peaked at or even exceeded 30 °C, and the coniferous forest in the border zone was the hottest in the same year (Fig. 2III).

In Hainich (Fig. 2III), no coniferous stands exist in the core zone. The year 2018 stood out as the warmest year with the highest average hot-day LST in the coniferous stands of border zones (app. 27.5 °C). Subsequently, all forest types experienced a decline in LST, converging to similar levels (app. 26 °C), except for the broad-leaved core, which saw a 1.3 °C rise. Unlike the trend observed in Grumsin, 2018 recorded a higher mean hot-day LST compared to 2022.

Throughout the observation period, in Jasmund where coniferous stands were absent, the broadleaved border zones displayed the higher average hot-day LST compared to all other zones. The year 2019 followed by 2022 were the hottest years for Jasmund (Fig. 2III). There was a temperature increase from 2017 to 2019, followed by a decrease until 2021. LST then rose again in 2022 before decreasing in 2023 (Fig. 2III).

Kellerwald (Fig. 2III) had no stark differences in mean hot-day LST across forest types and zones. Similar to Hainich, the year 2018 followed by 2019 stood out as the hottest years. Kellerwald forest experienced a steep warming on hot days from 2017 to 2018 (roughly 17.5 °C to almost 30 °C).

In Serrahn (Fig. 2III), the years 2019 and 2022 were the hottest years. The forests of the border zone were cooler in 2017 than in 2019. During the hottest year (i.e. 2019 and 2022), the buffer and border forests exhibited the same mean LST and were 2.5 °C cooler than core zones.

### Vitality change of forests in and around old-growth forests

We used 145 cloud-free days during the summer months (May-September) between 2017 and 2023 for the analysis of vitality and vitality change. The results of our Kruskal-Wallis test showed that there were significant differences in NDVI between different zones (χ² =25737, df = 2, *p* < 0.001). Furthermore, pairwise comparison shows the buffer had significantly higher NDVI than the border (z = 125.596, *p* < 0.001) and significantly lower NDVI than the core zone (z = 56.668, *p* < 0.001) (Table S4).

**Fig. 3:**
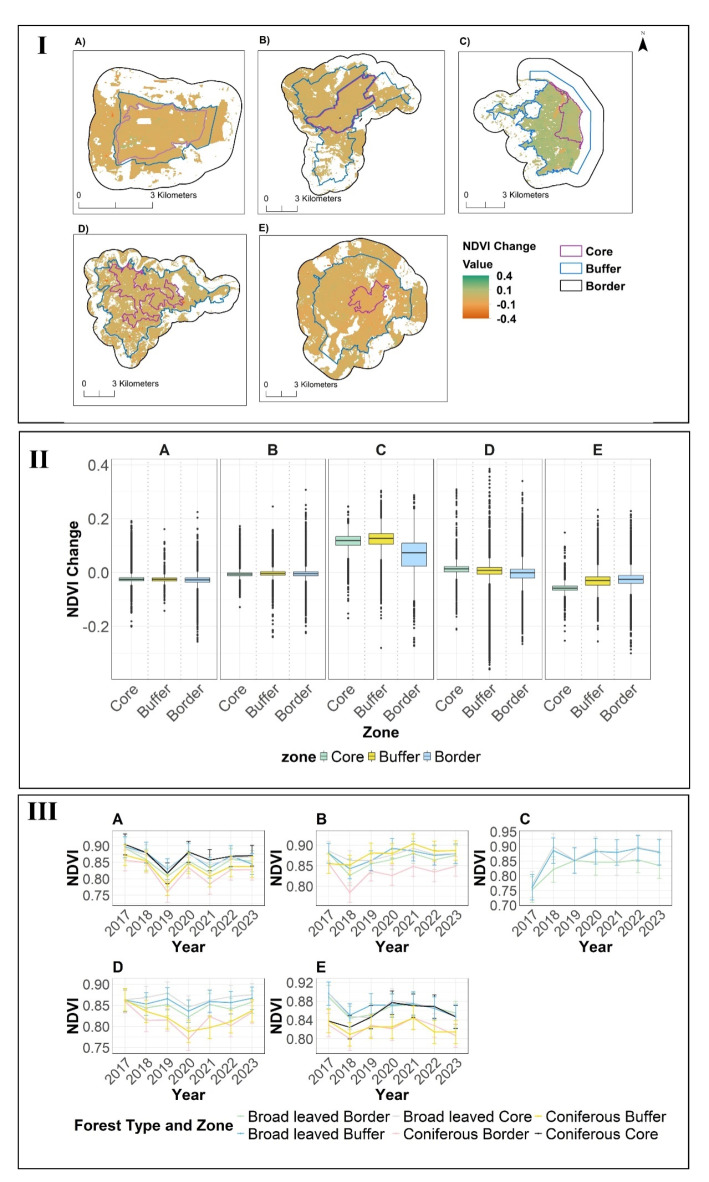
NDVI in German Component parts of World Heritage Beech Forests between 2017 and 2023; **A**) Grumsin, **B**) Hainich, **C**) Jasmund, **D**) Kellerwald and **E**) Serrahn using NDVI (Landsat 8,9). The NDVI is defined as “greenest” pixels (i.e., the highest NDVI value) of summer month composites (May-September) in the Landsat 8,9 between 2017–2023 for the core, buffer and border areas. (**I**) Map of recent vitality change (differences in NDVI of 2023 from 2017), (**II**) Recent vitality change (differences in vitality of 2023 from 2017) for the core, buffer and border zone forests (tree cover ≥ 30%), and (**III**) NDVI time series as mean of greenest pixels per year for the core, buffer and border zones and distinguished by forest types. Forests defined as tree cover greater than 30%.

Our findings on tree vitality based on NDVI show a comparable vitality in all three zones of Grumsin and Hainich based on greenest pixels in the summer months May to September from 2017 to 2023 (Fig. S1 and S2). In Jasmund, both buffer and core areas have higher NDVI than the border zone (Fig. S1 and S2). NDVI in the border zone of Kellerwald is lower than in the other zones. Serrahn’s core area boasts higher vitality than its surroundings (Fig. S1 and S2). During the study period from 2017 to 2023, the overall NDVI change in all three zones indicates that only Jasmund experienced a positive NDVI change across all zones (Fig. 3I).

Regarding the change of NDVI from 2017 to 2023, in Jasmund, both the core and buffer zones showed a positive change (+ 0.1 in core and buffer; +0.05 in border) in NDVI (Fig. 3II). Notably, in Serrahn, the core experienced a more substantial decrease (− 0.08) compared to the buffer and surroundings (Fig. 3II). For other component parts the NDVI change was 0 (for all zones of Hainich and Kellerwald) or near to 0 (for all zones of Grumsin) (Fig. 3II).

Throughout the observation period, the NDVI time series showed diverse patterns in NDVI trends with varying degrees of stability, recovery, and decline across core, buffer and surrounding zones in all five component parts.

In Grumsin, 2017 recorded highest NDVI whereas lowest NDVI was recorded in 2019. Likewise, there was an NDVI increase from 2019 to 2020 (Fig. 3III). Throughout the observation period, coniferous forests consistently showed lower NDVI values compared to broadleaved forests. Particularly, core coniferous forests consistently displayed higher NDVI values than broadleaved areas (Fig. 3III).

Hainich suffered a decline in 2018, followed by recovery and stability until 2023 without a subsequent increase. Coniferous buffer areas exhibited highest vitality after 2018 until 2023 (Fig. 3III).

In Jasmund, NDVI was lowest in 2017, followed by a steep increase from 2017 to 2018 and stabilization thereafter (Fig. 3III). Broadleaved core areas consistently exhibited higher NDVI values. Also, in Jasmund the core out-performed other zones (Fig. 3III).

Kellerwald experienced its years with lowest NDVI in 2020, with subsequent recovery. There was variation in forest types (lines far from each other), with broadleaved forests relatively stable while coniferous forest varied substantially. Core areas had a consistently higher NDVI than buffer and borders (Fig. 3III).

Serrahn suffered from a sharp NDVI decline in 2018, followed by stabilization and another decrease from 2021 onwards (Fig. 3III). Broadleaved forests consistently had higher NDVI values than coniferous forests. Over all, across all the five component parts, core areas showed higher NDVI followed by buffer areas (Fig. 3III).

### Relation between LST and AT in the core zone

**Fig. 4:**
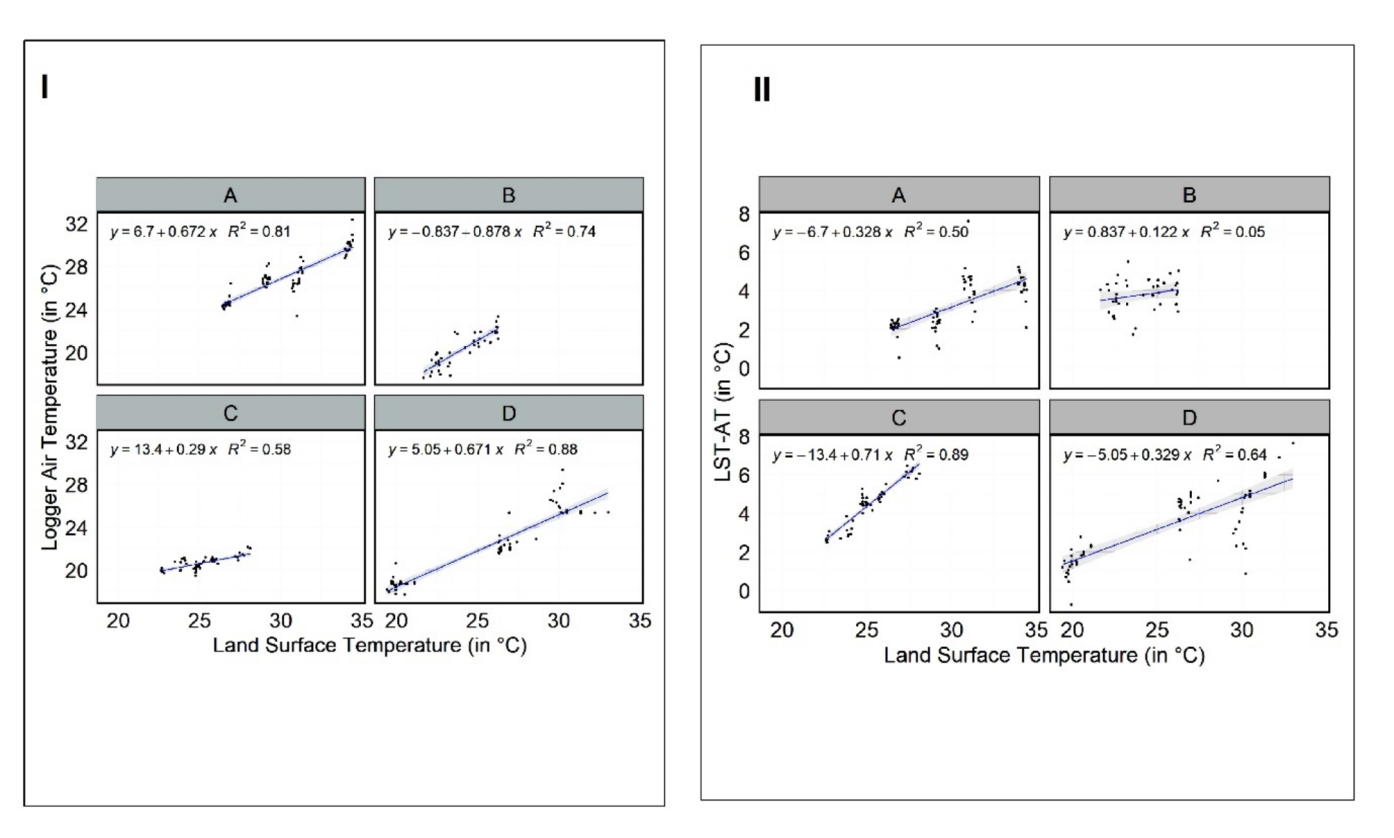
Temperature variations during the summer months of 2022 and 2023. (**I**) Relation between Landsat-derived Land Surface Temperature (LST) and Air Temperature (AT) from data loggers in the core zones; (**II**) Relation of LST and LST-AT difference in the core zones; **A**) Grumsin, **B**) Hainich, **C**) Kellerwald and **D**) Serrahn.

Our results of Spearman’s correlation tests between satellite-derived LST and AT from the data loggers in the dense old-growth forests of the core areas of four components—Grumsin, Hainich, Kellerwald, and Serrahn—reveal a strong positive monotonic relationship between LST and AT across all four study component parts (for Grumsin, ρ = 0.88, *p* < 0.001; for Hainich, ρ = 0.84, *p* < 0.001; for Kellerwald, ρ = 0.74, *p* < 0.001; for Serrahn, ρ = 0.89, *p* < 0.001). Furthermore, the regression analysis as illustrated in Fig. 4I further quantifies the relationship between LST and logger AT, demonstrating significant explanatory power (R²= 0.81 for Grumsin, R²= 0.74 for Hainich, R²= 0.58 for Kellerwald, and R²= 0.88 for Serrahn). Moreover, the regression equations suggest that for every 1 °C increase in LST, the logger AT is expected to increase by 0.67 °C at Grumsin, 0.88 °C at Hainich, 0.29 °C at Kellerwald, and 0.88 °C at Serrahn (Fig. 4I).

In addition, Spearman correlation tests between LST and the difference between LST and AT (LST-AT) show a positive relationship at the core of the component parts: ρ = 0.68 (*p* < 0.001) for Grumsin, ρ = 0.24 (*p* < 0.001) for Hainich, ρ = 0.92 (*p* < 0.001) for Kellerwald, and ρ = 0.81 (*p* < 0.001) for Serrahn. The regression analysis demonstrates varying explanatory power with R² values of 0.50 for Grumsin, 0.05 for Hainich, 0.89 for Kellerwald, and 0.64 for Serrahn (Fig. 4II). The regression equations suggest that for every 1 °C increase in LST, the temperature difference (LST-AT) increases by 0.32 °C in Grumsin, 0.12 °C in Hainich, 0.32 °C in Kellerwald, and 0.88 °C in Serrahn. While the relationship between LST and the temperature difference is strong in Grumsin, Kellerwald, and Serrahn, it is weak in Hainich.

## Discussion

### Micro and meso-climatic performance

Our time series analysis reveals that all the component parts were affected by extreme heat events during the study period. This is particularly evident during the extreme heat events of 2018 and 2022 where elevated LST were recorded consistently across all component parts. These findings coincide with the warm and dry periods observed by several studies in the study region^10,30,64,65^. Furthermore, results show a significant positive influence of years on hot days, except 2021, highlighting climate extreme days that becomes frequent with current global warming trends.

LST was found to vary differently over the years and zonation, indicating the role of zoning in determining the LST for that area. Our results confirm the effectiveness of buffer zones in regulating extreme-temperature during hot summer days, since the core and buffer zone are cooler than the border zonefor all years from 2017 to 2023 (Table S2). Although unmanaged beech forests are known to better withstand extreme temperature and climate stress with minimal effect in central Germany, our findings suggest that this resilience may only hold true for single occurrences of extreme summer years as claimed by Zimmerman et al.^66^. Our results are consistent with earlier studies that show the similar negative relationship between LST and forest vitality in the aftermath of the 2018, 2019 and 2020 summer heat^10,67–69^. Therefore, the vulnerability of beech forests to warming temperatures can possibly be attributed to cumulative stresses caused by the series of multi-year heat and drought conditions.

For all four component parts, our remotely sensed LST correlated well with in-situ measurements and revealed site-specific cooling effects of the forest canopies during hot summer days. Several previous studies also used satellite-derived LST data and the ground AT and found similar cooling trends under forest canopies^46,52,55,70^. In addition, we observed that the cooling effect in the forest interior increases with increasing landscape temperature (LST), implying that buffering of extreme temperatures becomes more effective at higher overall temperatures, which would be a meaningful ecological function of old-growth forests. However, the effect varies between components, indicating the complexity of temperature regulation dynamics in old-growth forests. Clearly, the temperature regulating capacity of forests is to some extent site-specific and would depend on the quality of both the forest and the surrounding matrix.

### Zoning effectiveness

Mostly, the core zones of the component parts are relatively resistant to heat stress and exhibit higher vitality and are also capable of post-extreme event recovery, namely NDVI increase after hot years. This could be attributed to their intact nature, past human disturbances (for example; military training grounds), dense canopy of beech, and higher connectivity in the core region compared to the surroundings^26,30,71^. However, it is important to acknowledge that the resilience and recovery potential of these core zones may be influenced by additional factors such as their size and characteristics of surrounding areas. Unfortunately, the remnant old-growth forests are very small patches surrounded by a mostly unfavourable matrix which impacts its overall resilience (Fig. S4).

Our study highlights the complexity of NDVI dynamics within forest ecosystems. While higher NDVI in core zones can indicate effective protection, it is not always related to old-growth or mature forests. For instance, in Grumsin, higher NDVI (or re-greening) in certain core areas is also due to growth of shrubs and young trees in storm-induced tree fall gaps, demonstrating the forest’s capacity for regeneration.

These observed NDVI dynamics are influenced by an interplay of various factors besides climate conditions, including soil properties, biodiversity and species distribution, topographical features such as elevation, slope and exposition, landscape fragmentation, historical and current forest management practices, and episodic disturbance events^26,72–74^.

There have been debates on which forest management approach – with no, high or low intensity intervention- has higher recovery rates^75–77^. Our findings are consistent with the most recent near-global assessment of naturally regenerating forests by Li et al.^78^ which pointed out the higher ecological recovery potential of unmanaged forest as compared to the managed forests and additionally claimed that the latter are more vulnerable to climate and human-induced stresses.

The appropriateness of buffer zones depends on the nature of the negative factors that have to be buffered. We conclude that effectiveness in reducing temperature extremes of buffer zones of the five component parts varies substantially with their size, management and geographical context influencing their performance. For instance, Grumsin, with rather narrow buffer zones, experiences higher temperatures in all zones and is less protected from heat, illustrating the need for larger buffer zones with continuous forest cover and high connectivity. This would reduce impacts of edge effects and disturbances to the core forest area. Our findings support the need for an adequately sized buffer zone, as climate change advances, it will become increasingly important to maintain and even strengthen the microclimatic buffering effect of the forest. A recent investigation by Mann et al.^26^ on the relation of forest fragmentation with temperature and vitality in Germany found that larger intact forests have relatively cooler temperatures on hot days and higher overall vitality. Hence, any opportunity to connect forest fragments should be prioritized^79^. While there are ongoing debates about expanding buffer zones due to concerns about reducing land available for agriculture or other land uses, larger buffer zones can help moderate temperature extremes, creating more stable conditions for crops and promoting better yields during adverse climate conditions by reducing forest fragmentation^80,81^.

Nevertheless, it is important to recognize the trade-offs involved, such as potential short-term economic impacts on agriculture-dependent communities and the challenge of reallocating land. A balance between conservation and agricultural needs is essential through careful land-use planning to ensure both forest conservation and agricultural productivity. In the long run, these strategies could foster a more resilient landscape that supports sustainable farming while safeguarding protected areas.

Similarly, the influence of geographical context, such as proximity to water bodies plays a role. Jasmund, which has a coastal boundary, clearly benefits from cooling protection provided by the sea, resulting in cooler forest temperatures during the hot days compared to other component parts in north-eastern Germany. This outcome is to be expected as water bodies are generally acknowledged to have stronger and extensive cooling effects than any land features^19^. Also, the cooling intensity is strongest in summer, since evaporation in the water bodies is higher in summer^82–86^.

### Uncertainty and influencing factors

One uncertainty arises from whether the cooling effect of old-growth forests is significantly different between hot (LST > 30 °C) and cooler (LST < 30 °C) days. The model results support that old-growth forests possess a significant temperature regulation capacity. On cooler days this cooling effect is less pronounced (Table S5). While the hot-days consistently demonstrated significant cooling effects across locations (component parts) and zones (Table S1), the cooler days indicated that several locations had non-significant effects across different component parts (Table S5). This discrepancy suggests that the cooling effectiveness of old-growth forests may depend on specific temperature thresholds. Several previous studies including Norris et al.^34^ and Li et al.^54^ also found significant differences in the ability of the forest stands regulate temperature became apparent at higher temperature thresholds. Therefore, it is reasonable to consider hot days when assessing the cooling capacity of forests.

When analysing the cooling effect of zoning, some methodological limitations of our study have to be taken into account. Firstly, for technical reasons, we could not include Jasmund in the in-situ measurements. Given its coastal location, future research should investigate how the cooling effect of forests differs in such environments. Secondly, we measured AT only in the core areas with dense old-growth forests. The buffer and border areas were not included in the in-situ measurements. Thirdly, the number of summer days studied for the correlation of AT and LST was constrained by the availability of Landsat data due to the lengthy revisit time of the satellite every 16 days.

## Conclusions and recommendations for management

Overall, the German component parts of UNESCO World Heritage beech forests, on hot days, regulate temperatures relatively well, but have all been affected by the recent heat events. Clearly, concrete temperature and vitality signatures are rather individual and are strongly influenced by landscape characteristics and the surrounding matrix. Although some of the forests were severely affected by severe drought and heat, and there are clear signs of (temporary) reductions in vitality, partly caused by increased tree mortality, it is important to note that there was no collapse of entire beech stands, yet. We therefore conclude that if more of Germany’s forests were broadleaved and mature (or even old-growth), we would not be lamenting the loss of forest cover that we see in conifer plantations. The recent German ‘forest crises would be less pronounced if there were more old-growth and mature forests.

Furthermore, we assume that forest health would be even better if there were better connectivity and higher forest cover in some critical regions. The resilience performance and provision of regulating ecosystem services would also have been positively influenced by more older forests. For the sake of forest ecosystem health, but also to ensure future delivery of key regulating ecosystem services for human well-being, and to provide living laboratories for management, older forests need to be conserved. We recommend increasing the area of old-growth and self-regulating forests, i.e., to include larger buffer areas around old-growth and mature forests in the conservation regime, and to establish forest corridors between unconnected forest patches. It is also commendable to create a mosaic in all managed forest landscapes that includes unmanaged areas that have the opportunity to develop into old-growth ecosystems.

The UNESCO World Heritage component parts are flagships of forest conservation and provide valuable insights into the functioning of ecosystems that have not been interfered with by humans in recent times. We argue that comprehensive, standardized and systematic monitoring, using both on-site and remote sensing methods, will continue to be an appropriate means of better understanding and documenting their performance, including in comparison to heavily managed landscapes. We emphasize that the conservation of the component parts of the UNESCO World Heritage must take into account both their intrinsic characteristics and their function in the wider landscape context. The effectiveness of their protection may depend on the performance of ecosystems both within and outside the component parts.

## Electronic supplementary material

Below is the link to the electronic supplementary material.

Supplementary Material 1

Supplementary Material 2

Supplementary Material 3

Supplementary Material 4

Supplementary Material 5

Supplementary Material 6

## Data Availability

The datasets generated during and/or analysed during the current study are available from the corresponding author on reasonable request.
