# Supplementary material for: Old-growth beech forests in Germany as cool islands in a warming landscape
Source: Sci Rep. 2024 Dec 5;14:30311. doi: 10.1038/s41598-024-81209-0 (PMC11621415; doi:10.1038/s41598-024-81209-0)
Supplement: Supplementary file 1 — Supplementary Material 1 [file 41598_2024_81209_MOESM1_ESM.docx]

**Old-growth beech forests in Germany as cool islands in a warming landscape**

Yojana Adhikari^1,*^, Nadine Bachstein^1^, Charlotte Gohr^1,2^, Jeanette S. Blumröder^1^, Caroline Meier^1^, Pierre L. Ibisch^1^

^1^Centre for Econics and Ecosystem Management, Eberswalde University for Sustainable Development, Alfred-Möller-Str. 1, 16225 Eberswalde, Germany

^2^ Center of Methods and Faculty of Sustainability, Leuphana University; Lüneburg, 21335, Germany

***** Yojana.Adhikari@hnee.de

**Supplementary Information**

Table S1: Results of linear mixed effect model i.

Table S1 presents the results for model i. *LST ~ Forest Cover * Location + NDVI + (1|Location/Zone).* The model explains 48.1% of the variance in the fixed effects, which include forest cover, location, and NDVI. For the random effects associated with location and zonation, the model accounts for 74.9% of the variance. A hot-day was defined as one where the maximum LST reached or exceeded 30°C in any forest pixel in Germany.

|  | **LST for hot-days**  **(**Per pixel mean of LST>30°C in any forest**)** | | |
| --- | --- | --- | --- |
| *Predictors* | *Estimates* | *CI* | *p* |
| (Intercept) | 39.92 | 38.84 – 40.99 | **<0.001** |
| Forest cover [Coniferous forest] | 0.55 | 0.48 – 0.62 | **<0.001** |
| Location [Hainich] | -3.34 | -4.86 – -1.82 | **<0.001** |
| Location [Jasmund] | -4.38 | -5.90 – -2.86 | **<0.001** |
| Location [Kellerwald] | -2.11 | -3.63 – -0.59 | **0.006** |
| Location [Serrahn] | -2.00 | -3.52 – -0.48 | **0.010** |
| NDVI | -12.70 | -12.80 – -12.60 | **<0.001** |
| Forest cover [Coniferous forest] *Location [Hainich] | -0.38 | -0.46 – -0.29 | **<0.001** |
| Forest cover [Coniferous forest] * Location [Kellerwald] | -0.14 | -0.22 – -0.06 | **0.001** |
| Forest cover [Coniferous  forest] * Location  [Serrahn] | -1.01 | -1.08 – -0.93 | **<0.001** |
| **Random Effects** | | | |
| σ^2^ | 0.78 | | |
| τ_00_ _Zone:Location_ | 0.79 | | |
| τ_00_ _Location_ | 0.04 | | |
| ICC | 0.52 | | |
| N _Zone_ | 3 | | |
| N _Location_ | 5 | | |
| Observations | 320425 | | |
| Marginal R^2^ / Conditional R^2^ | 0.481 / 0.749 | | |

Table S2: Results of linear mixed effect model ii.

Table S2 presents the results of the linear mixed-effects model ii. *LST ~ NDVI * Zone + Year + (1|Location/Year).* The model explains 41.5% of the variance for the fixed effects, which include NDVI, zone interactions, and year. For the random effects associated with location and year, the model accounts for 89.1% of the variance. A hot-day was defined as one where the maximum LST reached or exceeded 30°C in any forest pixel in Germany.

|  | **LST for hot-days**  **(**Per pixel mean of LST>30°C in any forest**)** | | |
| --- | --- | --- | --- |
| *Predictors* | *Estimates* | *CI* | *p* |
| (Intercept) | 23.42 | 21.30 – 25.54 | **<0.001** |
| NDVI | -5.05 | -5.43 – -4.66 | **<0.001** |
| NDVI:Zone buffer | -4.63 | -5.05 – -4.22 | **<0.001** |
| NDVI:Zone border | -5.82 | -6.26 – -5.38 | **<0.001** |
| Year2018 | 4.61 | 2.60 – 6.63 | **<0.001** |
| Year2019 | 5.97 | 3.95 – 7.99 | **<0.001** |
| Year2020 | 2.76 | 0.74 – 4.77 | **0.007** |
| Year2021 | 2.10 | 0.08 – 4.12 | **0.041** |
| Year2022 | 6.57 | 4.56 – 8.59 | **<0.001** |
| Year2023 | 4.64 | 2.62 – 6.66 | **<0.001** |
| buffer | 4.16 | 3.80 – 4.53 | **<0.001** |
| border | 5.59 | 5.22 – 5.97 | **<0.001** |
| **Random Effects** | | | |
| σ^2^ | 1.30 | | |
| τ_00_ _Year:Location_ | 2.65 | | |
| τ_00_ _Location_ | 3.06 | | |
| ICC | 0.81 | | |
| N _Year_ | 7 | | |
| N _Location_ | 5 | | |
| Observations | 128713 | | |
| Marginal R^2^ / Conditional R^2^ | 0.415 / 0.891 | | |

Table S3: Results of linear mixed effect model iii.

Table S3 presents the results of the linear mixed effect model iii. *LST ~ Year * Zone + NDVI + (1|Location/Year).* The model accounts for 42.7% of the variance in the fixed effects, including year, zone interactions, and NDVI. For the random effects related to location and year, the model explains 89.8% of the variance. A hot-day was defined as one where the maximum LST reached or exceeded 30°C in any forest pixel in Germany.

|  | **LST for hot-days**  **(**Per pixel mean of LST>30°C in any forest**)** | | |
| --- | --- | --- | --- |
| *Predictors* | *Estimates* | *CI* | *p* |
| (Intercept) | 28.24 | 26.15 – 30.32 | **<0.001** |
| NDVI | -9.55 | -9.68 – -9.42 | **<0.001** |
| Year2018 | 3.55 | 1.53 – 5.56 | **0.001** |
| Year2018:Zone border | 0.32 | 0.25 – 0.38 | **<0.001** |
| Year2018:Zone buffer | 2.00 | 1.95 – 2.06 | **<0.001** |
| Year2019 | 4.88 | 2.86 – 6.89 | **<0.001** |
| Year2019:Zone border | 0.58 | 0.51 – 0.65 | **<0.001** |
| Year2019:Zone buffer | 1.88 | 1.82 – 1.94 | **<0.001** |
| Year2020 | 1.72 | -0.29 – 3.74 | 0.094 |
| Year2020:Zone border | 0.54 | 0.47 – 0.61 | **<0.001** |
| Year2020:Zone buffer | 1.83 | 1.77 – 1.89 | **<0.001** |
| Year2021 | 1.36 | -0.65 – 3.38 | 0.184 |
| Year2021:Zone border | 0.07 | -0.00 – 0.13 | 0.064 |
| Year2021:Zone buffer | 1.45 | 1.40 – 1.51 | **<0.001** |
| Year2022 | 5.31 | 3.29 – 7.32 | **<0.001** |
| Year2022:Zone border | 1.07 | 1.00 – 1.14 | **<0.001** |
| Year2022:Zone buffer | 2.03 | 1.97 – 2.09 | **<0.001** |
| Year2023 | 3.51 | 1.50 – 5.52 | **0.001** |
| Year2023:Zone border | 0.71 | 0.64 – 0.78 | **<0.001** |
| Year2023:Zone buffer | 1.93 | 1.87 – 1.99 | **<0.001** |
| border | 0.12 | 0.07 – 0.17 | **<0.001** |
| buffer | -1.45 | -1.49 – -1.41 | **<0.001** |
| **Random Effects** | | | |
| σ^2^ | 1.22 | | |
| τ_00_ _Year:Location_ | 2.64 | | |
| τ_00_ _Location_ | 3.00 | | |
| ICC | 0.82 | | |
| N _Year_ | 7 | | |
| N _Location_ | 5 | | |
| Observations | 128713 | | |
| Marginal R^2^ / Conditional R^2^ | 0.427 / 0.898 | | |

Table S4: Dunn’s Test Pairwise Comparisons for zones

| Comparison | Z-value | p-value |
| --- | --- | --- |
| core-buffer | 56.668 | <0.001 |
| border-buffer | -125.597 | <0.001 |
| core-border | 153.497 | <0.001 |

(*Positive z value indicate higher NDVI in the first zone*)

Table. S5 Results of linear mixed effect model on non-hot days (LST < 30°C)

Table S5 presents the results of the linear mixed effect model: *LST ~ Forest_cover* Location + NDVI + (1|Location/Zone).* The model accounts for 22.1% of the variance in the fixed effects, including forest cover, location, and NDVI. For the random effects related to location and zone, the model explains 87.3% of the variance.

|  | **LST for non-hot days**  **(**Per pixel mean of LST<30°C in any forest**)** | | |
| --- | --- | --- | --- |
| *Predictors* | *Estimates* | *CI* | *p* |
| (Intercept) | 20.38 | 16.44 – 24.33 | **<0.001** |
| Coniferous | 0.43 | 0.36 – 0.49 | **<0.001** |
| LocationHainich | -3.91 | -9.49 – 1.67 | 0.169 |
| LocationJasmund | -2.17 | -7.75 – 3.40 | 0.445 |
| LocationKellerwald | -3.23 | -8.81 – 2.35 | 0.256 |
| LocationSerrahn | -2.45 | -8.02 – 3.13 | 0.390 |
| Forest_coverConiferous:LocationHainich | -0.33 | -0.41 – -0.26 | **<0.001** |
| Forest_coverConiferous:LocationKellerwald | -0.64 | -0.71 – -0.57 | **<0.001** |
| Forest_coverConiferous:LocationSerrahn | -0.66 | -0.73 – -0.59 | **<0.001** |
| NDVI | -4.04 | -4.13 – -3.95 | **<0.001** |
| **Random Effects** |  |  |  |
| σ^2^ 0.82 | | | |
| τ_00_ _Zone:Location_ | 0.27 | | |
| τ_00_ _Location_ | 3.96 | | |
| ICC | 0.84 | | |
| N _Zone_ | 3 | | |
| N _Location_ | 5 | | |
| Observations | 290528 | | |
| Marginal R^2^ / Conditional R^2^ | 0.221 / 0.873 | | |


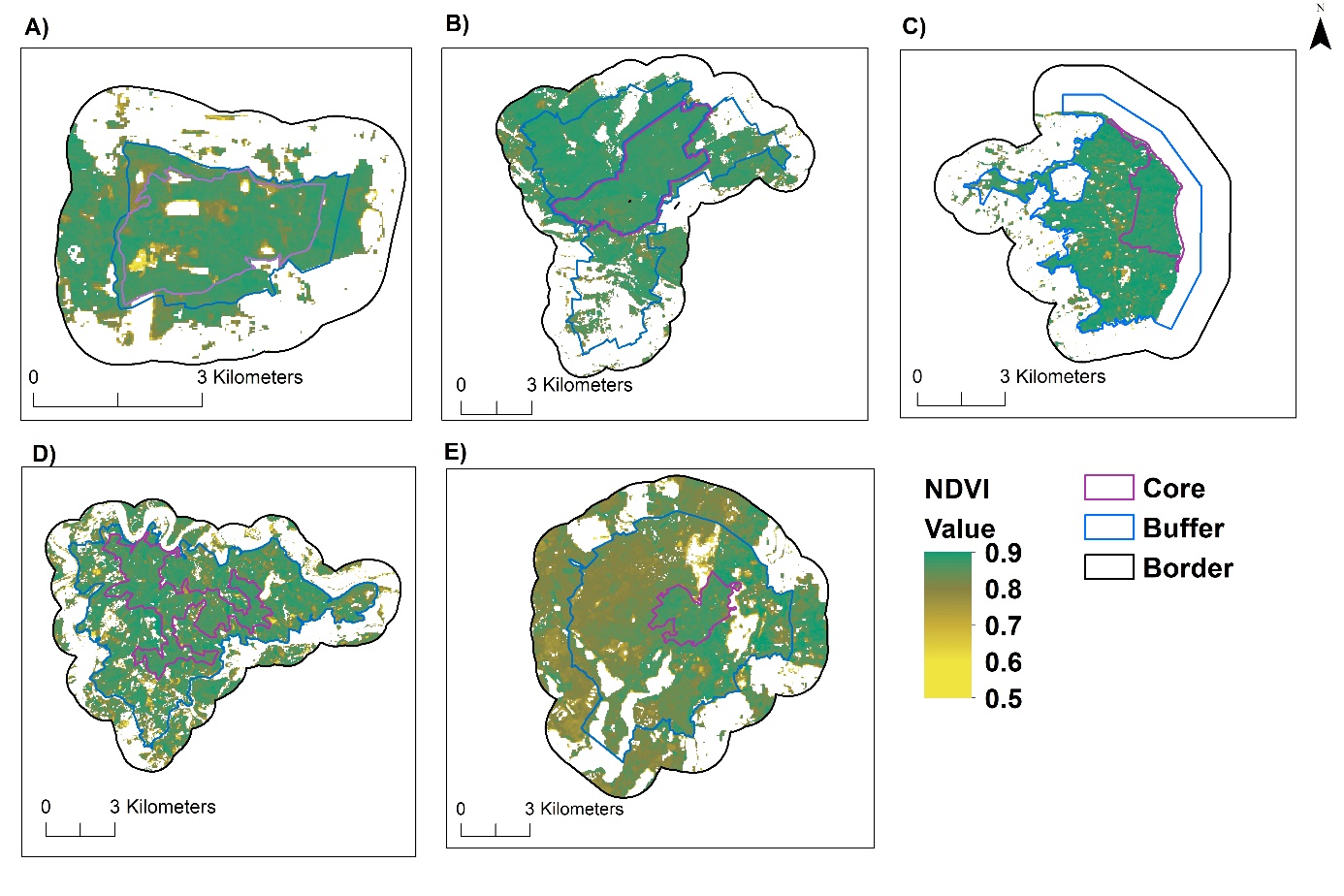


Fig. S1:  Recent average NDVI of the five WHB. The NDVI is defined as “greenest” pixels (i.e., the highest NDVI value) of summer month composites (May-September) in the Landsat 8,9 NDVI time series 2017-2023.


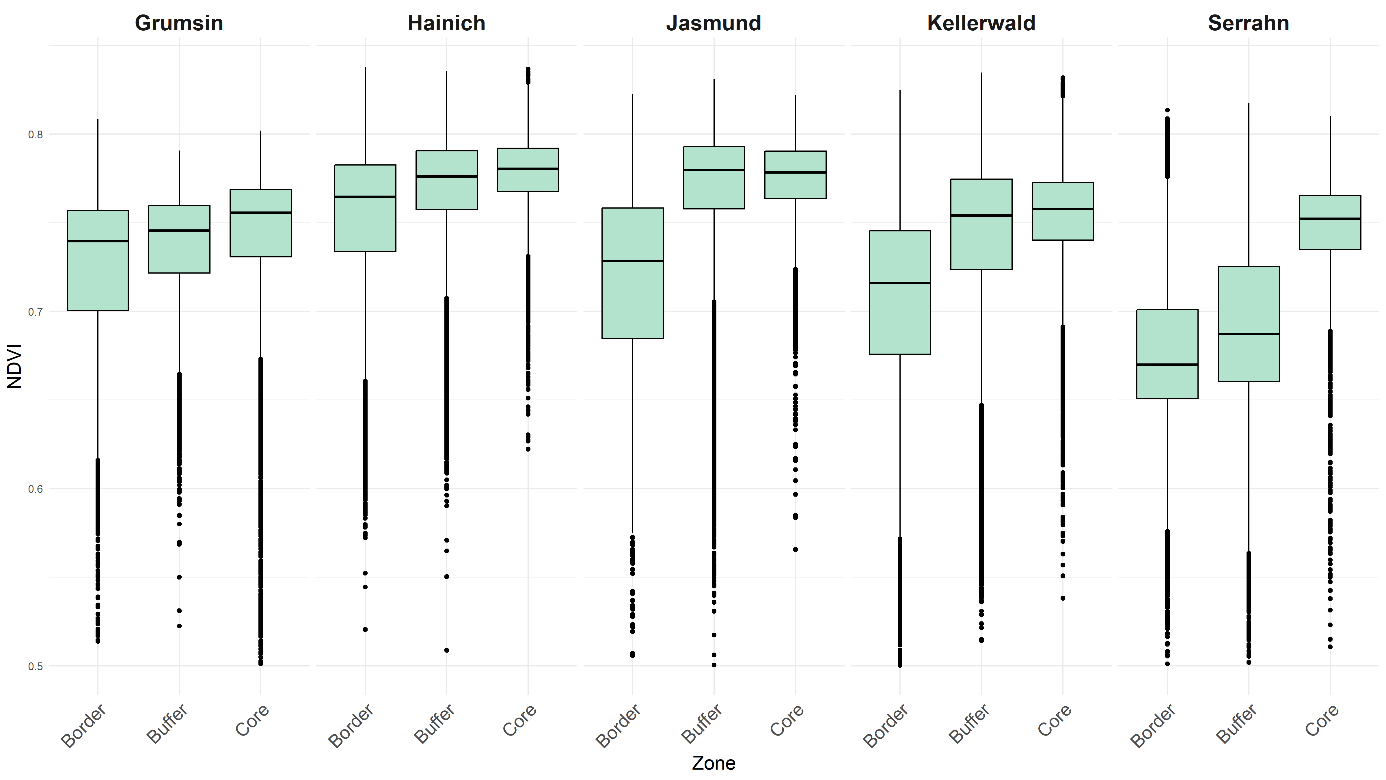


Fig. S2: Recent average NDVI for the core, buffer and border zones in the five German World Heritage beech forests. The NDVI is defined as “greenest” pixels (i.e., the highest NDVI value) of summer month composites (May-September) in the Landsat 8,9 NDVI time series 2017–2023 for the core, buffer and border areas.


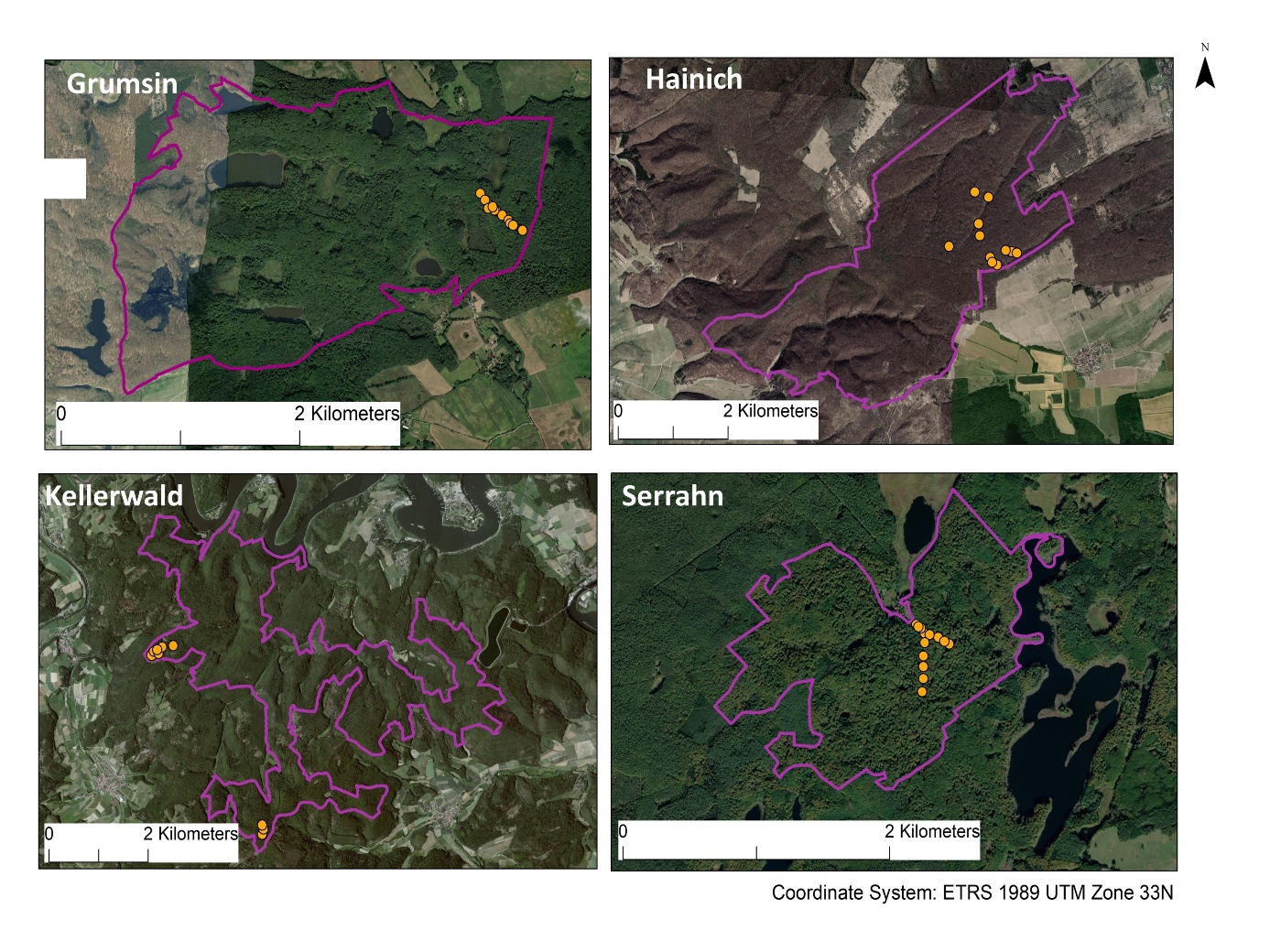


Fig. S3: Location of the microclimatic data loggers in the core zone of Grumsin, Hainich, Kellerwald and Serrahn World Heritage beech forests used for quantifying the differences between LST and AT.


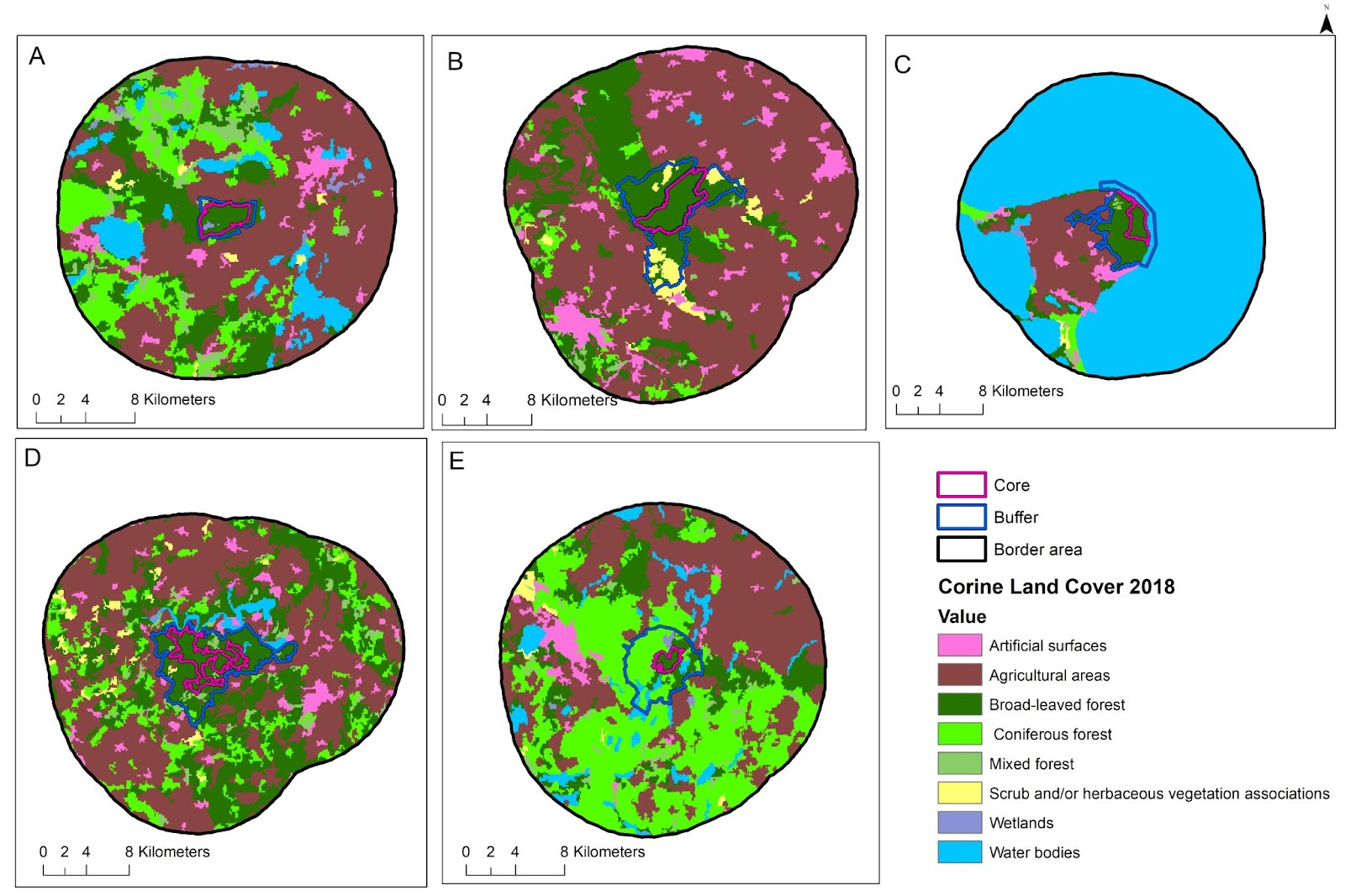


Fig. S4: Land Use Map based on the CORINE Classification (^1^), showing the zoning of the five German sites with core zones in pink, buffer zones in blue, and borders in black (extending 10 km from the buffer zone) for A) Grumsin, B) Hainich, C) Jasmund, D) Kellerwald, and E) Serrahn.


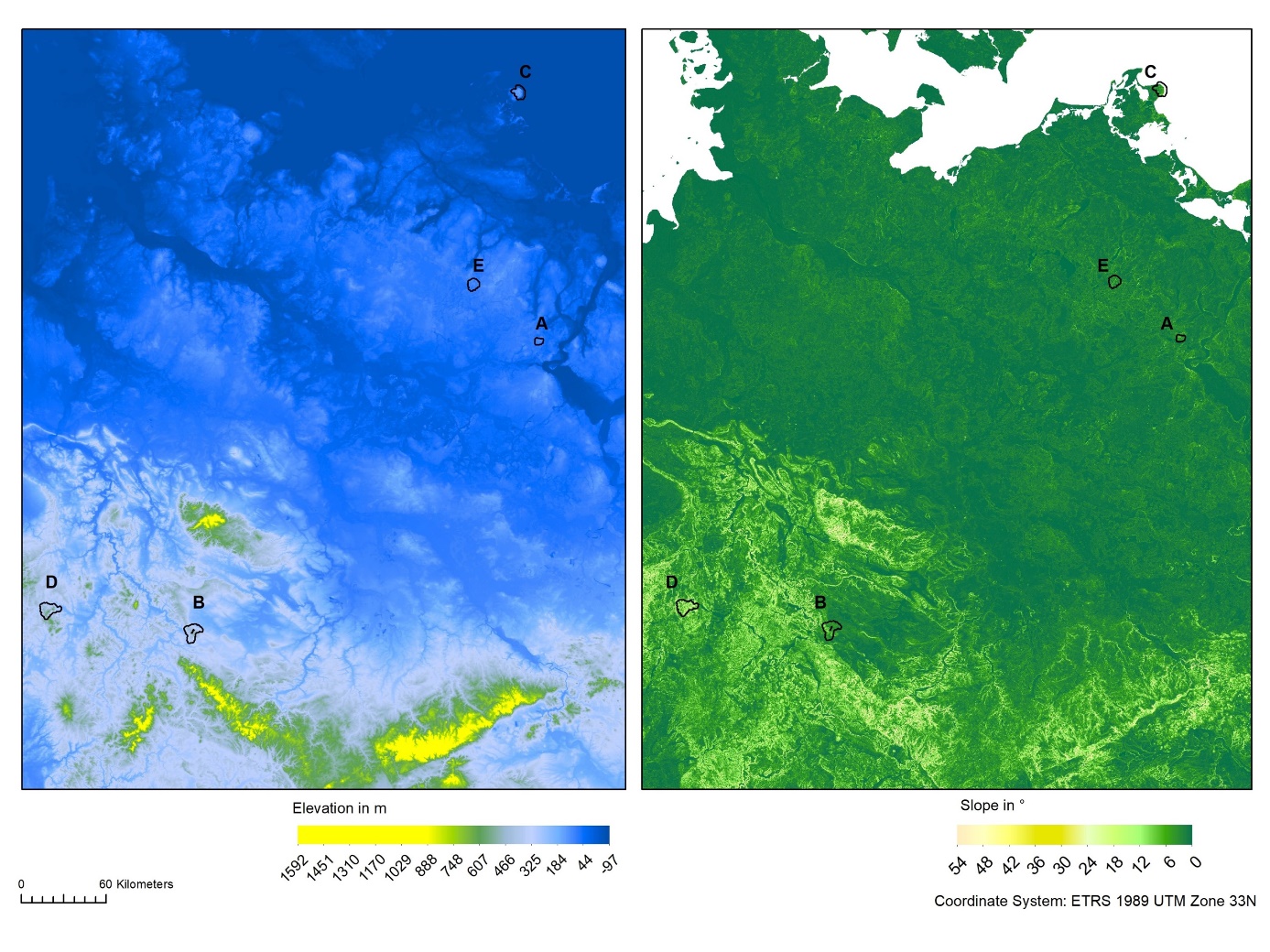


Fig. S5: Elevation and Slope of the German World Heritage Beech Forests: A) Grumsin, B) Hainich, C) Jasmund, D) Kellerwald, and E) Serrahn.

References

1. European Environment Agency. CORINE Land Cover 2018 (raster 100 m), Europe, 6-yearly - version 2020_20u1, May 2020. European Environment Agency https://doi.org/10.2909/960998C1-1870-4E82-8051-6485205EBBAC (2019).
