# Supplementary figures and images for: Old-growth beech forests in Germany as cool islands in a warming landscape
Source: Sci Rep. 2024 Dec 5;14:30311. doi: 10.1038/s41598-024-81209-0 (PMC11621415; doi:10.1038/s41598-024-81209-0)

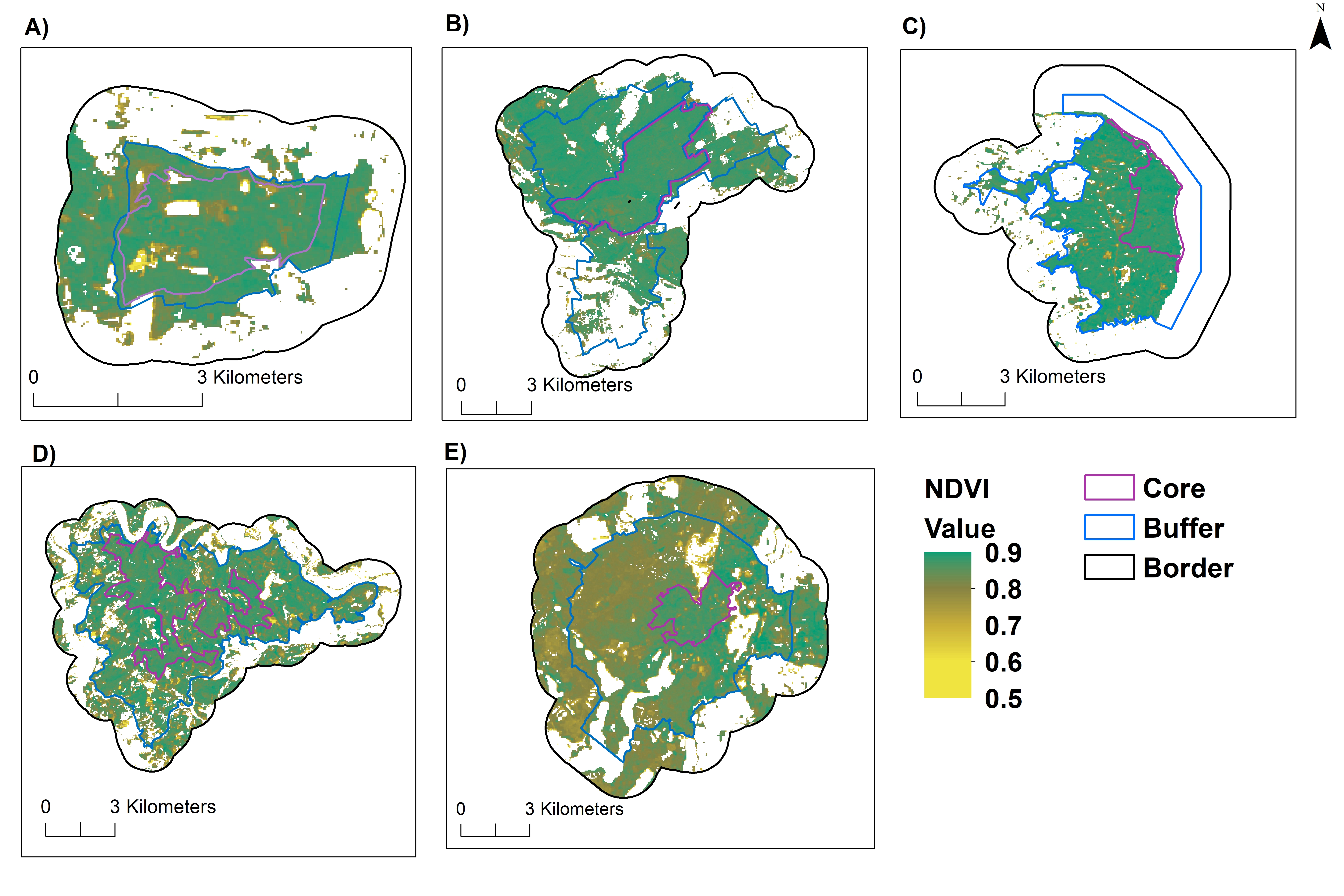

Supplement: Supplementary file 2 — Supplementary Material 2 [file 41598_2024_81209_MOESM2_ESM.jpg]

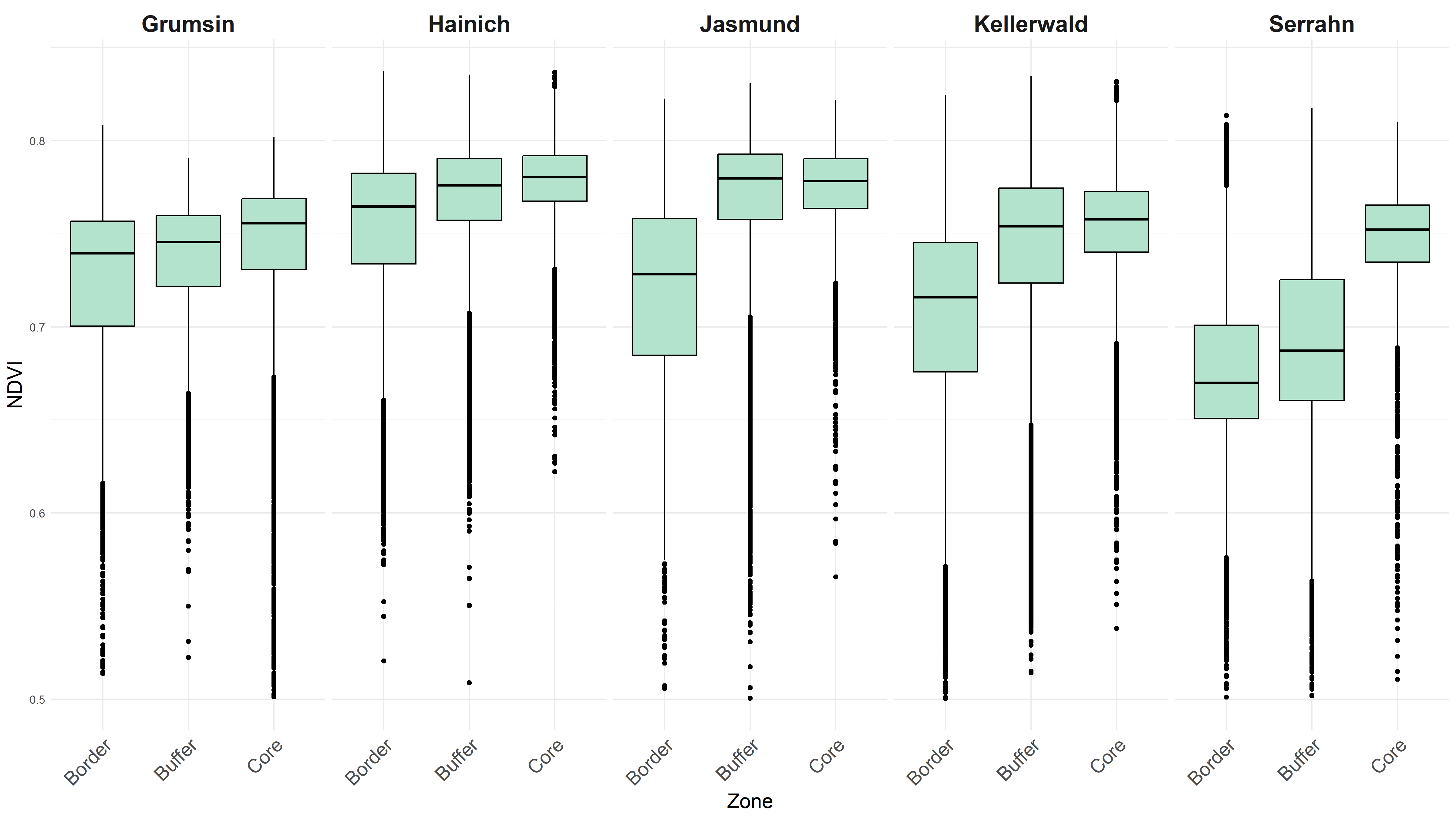

Supplement: Supplementary file 3 — Supplementary Material 3 [file 41598_2024_81209_MOESM3_ESM.tiff]

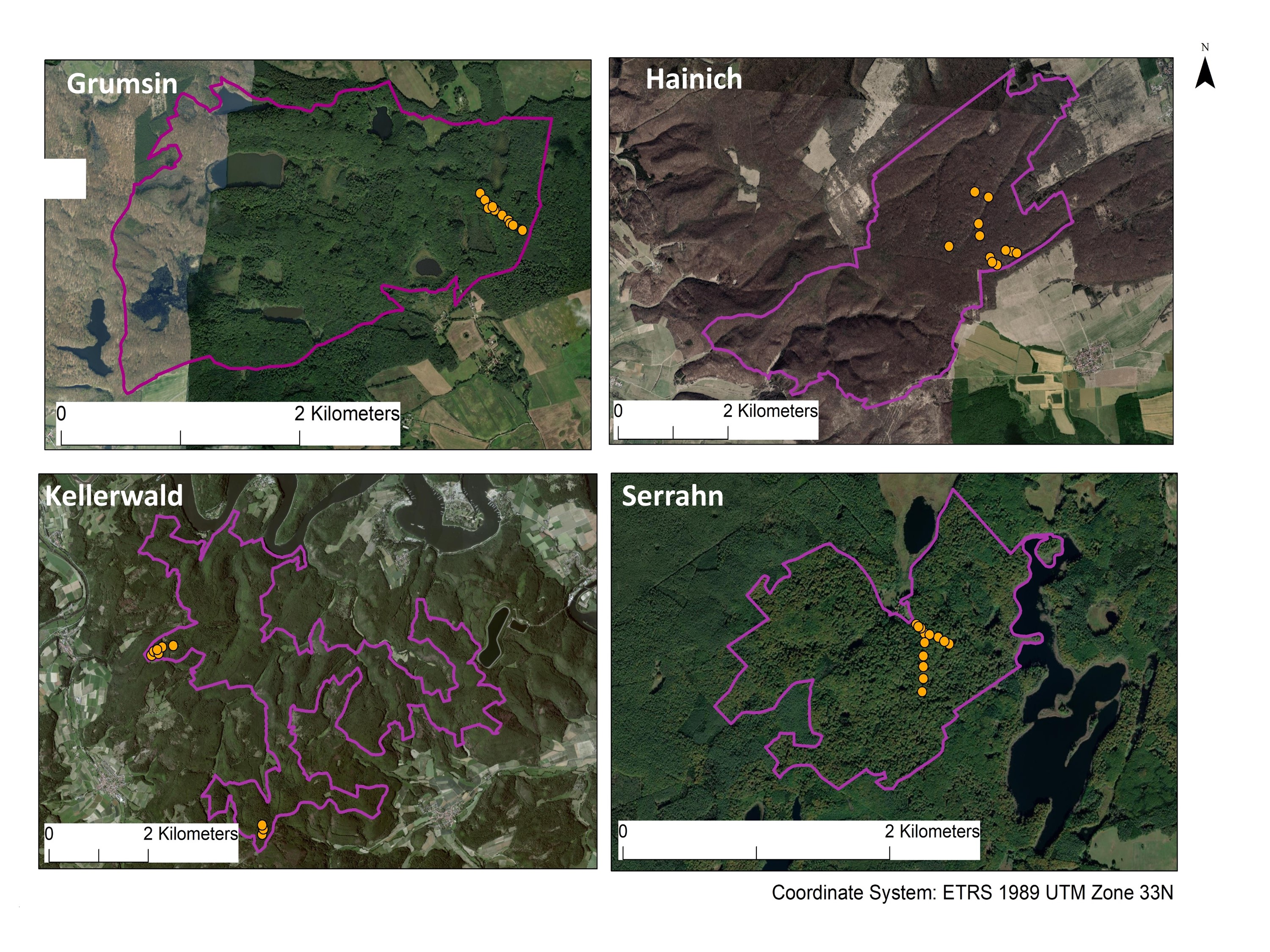

Supplement: Supplementary file 4 — Supplementary Material 4 [file 41598_2024_81209_MOESM4_ESM.jpg]

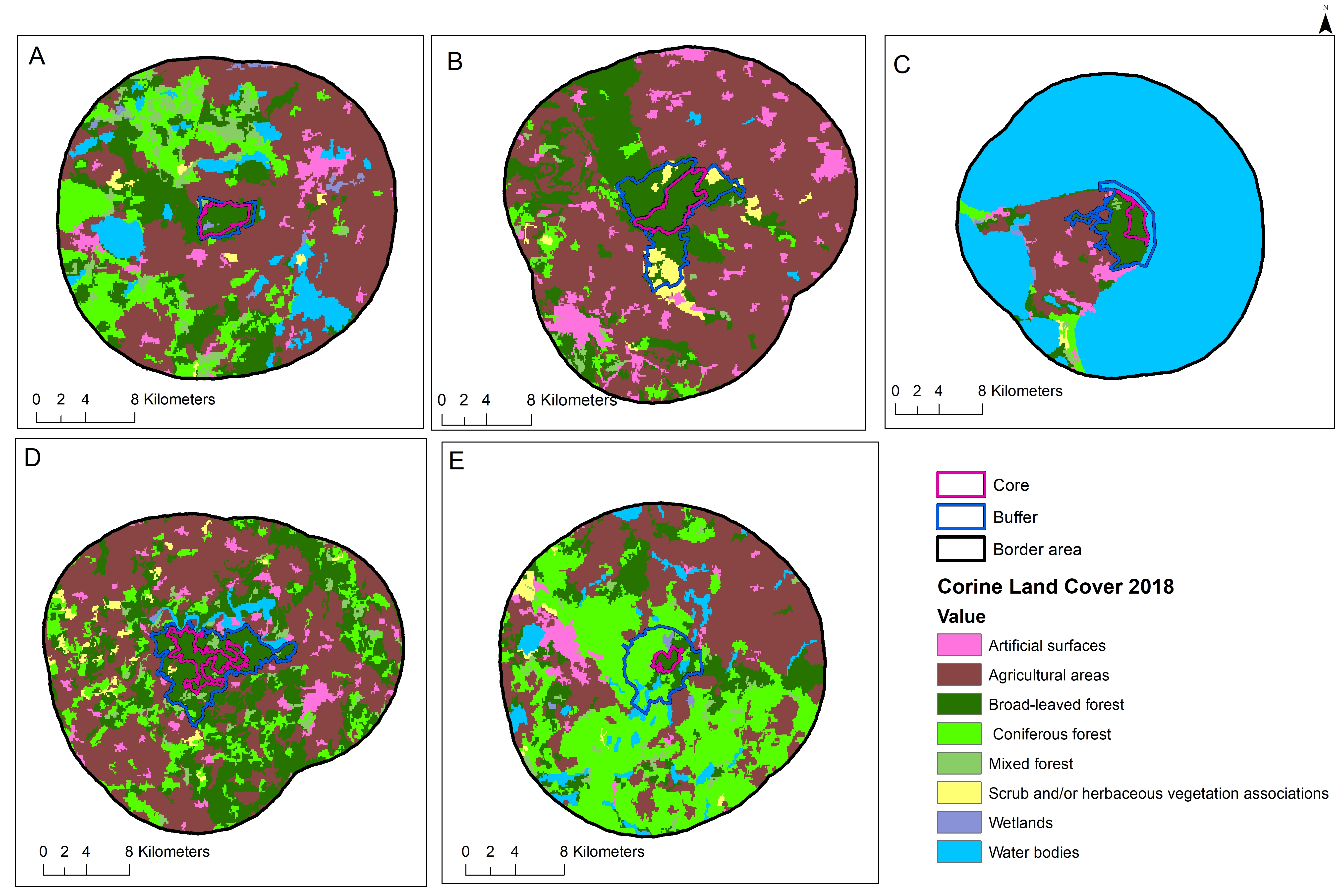

Supplement: Supplementary file 5 — Supplementary Material 5 [file 41598_2024_81209_MOESM5_ESM.jpg]

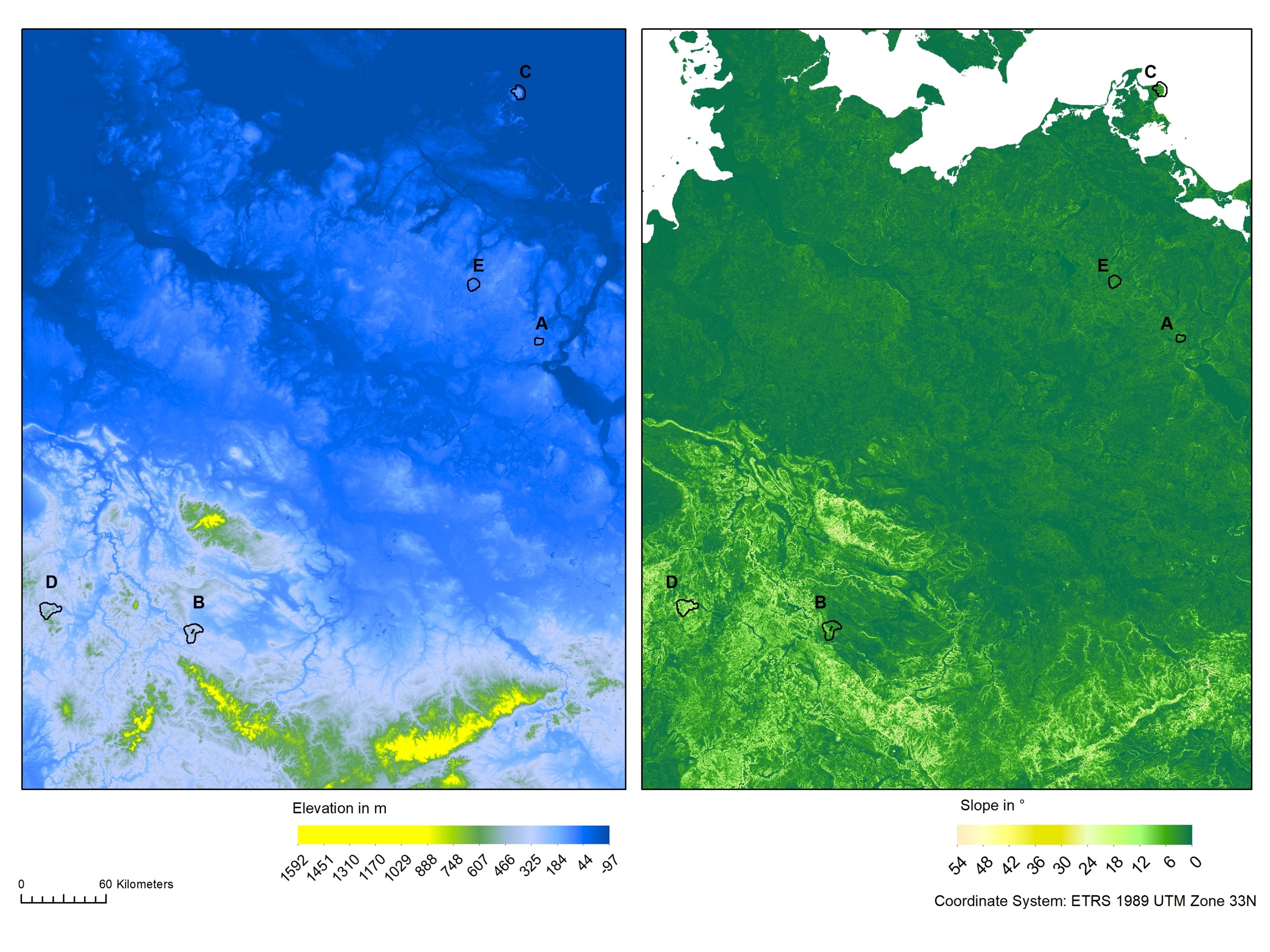

Supplement: Supplementary file 6 — Supplementary Material 6 [file 41598_2024_81209_MOESM6_ESM.jpg]
